# Genomic chart guiding embryonic stem cell cardiopoiesis

**DOI:** 10.1186/gb-2008-9-1-r6

**Published:** 2008-01-09

**Authors:** Randolph S Faustino, Atta Behfar, Carmen Perez-Terzic, Andre Terzic

**Affiliations:** 1 Marriott Heart Disease Research Program, Division of Cardiovascular Diseases, Departments of Medicine, Molecular Pharmacology and Experimental Therapeutics, and Medical Genetics, Mayo Clinic, First Street SW, Rochester, Minnesota 55905, USA; 2 Department of Physical Medicine and Rehabilitation, Mayo Clinic, First Street SW, Rochester, Minnesota 55905, USA

## Abstract

**Background:**

Embryonic stem cells possess a pluripotent transcriptional background with the developmental capacity for distinct cell fates. Simultaneous expression of genetic elements for multiple outcomes obscures cascades relevant to specific cell phenotypes. To map molecular patterns critical to cardiogenesis, we interrogated gene expression in stem cells undergoing guided differentiation, and defined a genomic paradigm responsible for confinement of pluripotency.

**Results:**

Functional annotation analysis of the transcriptome of differentiating embryonic stem cells exposed downregulated components of DNA replication, recombination and repair machinery, cell cycling, cancer mechanisms, and RNA post-translational modifications. Concomitantly, cardiovascular development, cell-to-cell signaling, cell development and cell movement were upregulated. These simultaneous gene ontology rearrangements engaged a repertoire switch that specified lineage development. Bioinformatic integration of genomic and gene ontology data further unmasked canonical signaling cascades prioritized within discrete phases of cardiopoiesis. Examination of gene relationships revealed a non-stochastic network anchored by integrin, WNT/β-catenin, transforming growth factor β and vascular endothelial growth factor pathways, validated by manipulation of selected cascades that promoted or restrained cardiogenic yield. Moreover, candidate genes within anchor pathways acted as nodes that organized correlated expression profiles into functional clusters, which collectively orchestrated and secured an overall cardiogenic theme.

**Conclusion:**

The present systems biology approach reveals a dynamically integrated and tractable gene network fundamental to embryonic stem cell specification, and represents an initial step towards resolution of a genomic cardiopoietic atlas.

## Abstract 2

Gene expression analysis of embryonic stem cells undergoing guided cardiogenic differentiation reveals the molecular fingerprint for committing to cardiac cell fate.

## Background

Expression patterns characterize the production and proliferation of stem cells [1,2]. In particular, unique genetic profiles are concealed in the rich pluripotent transcriptional background of embryonic stem cells and support their inherent potential for multiple and diverse cell fates [3-6]. Genome-wide profiling and system analyses, used to distinguish markers identifying stemness [7,8], and high-throughput approaches applied to categorize large scale transcriptional dynamics during stem cell development and specification provide an initial insight into the global genomics evolving in response to inductive stimuli [2,9,10]. Beyond identification of stemness markers, however, integration of genes promoting tissue-restricted differentiation becomes a priority [11,12]. Mapping genetic relationships underlying metamorphosis of a pluripotent into a monopotent stem cell would allow for directional control over developmental fate, enhancing targeted derivation of phenotype-specified cell types.

Indeed, the broad potential for regenerative therapy based on embryonic stem cell technology is hampered by the threat of neoplastic transformation associated with unsupervised pluripotency, mandating unipotential commitment prior to application [13,14]. A case in point is the need to secure controlled cardiogenesis of embryonic stem cells for safe heart repair [15-17]. Guided pro-cardiac programming has been established as a strategy to suppress the risk for uncontrolled tumorigenic growth outside the natural milieu of a developing embryo [18]. Cardiopoietic induction allowed activation of the cardiac program on a monolayer of stem cells, eliminating the confounding contribution of trigerminal differentiation [18,19]. Privileged access to the cardiac transcriptional program, otherwise camouflaged within the stem cell genomic background [20,21], provides an opportunity to selectively examine gene interrelationships vital for pluripotent streamlining into cardiopoiesis.

Here, a transcriptome profiling and tandem network analysis of embryonic stem cells during guided cardiogenic differentiation identified a molecular fingerprint, synthesized from an ontological functional switch, that commits the cells to a cardiac fate. Pathway prioritization of signaling axes during cardiopoiesis resolved a non-stochastic organization of genes underlying cardiac specification. Manipulation of high-priority nodes within this deconvoluted pro-cardiac gene network commanded cardiomyocyte derivation from primordial stem cells, demonstrating a responsive program amenable to molecular calibration during directed cardiogenesis.

## Results

### Distinct transcriptomes define transitions in stem cell cardiogenic restriction

**Figure 1:**
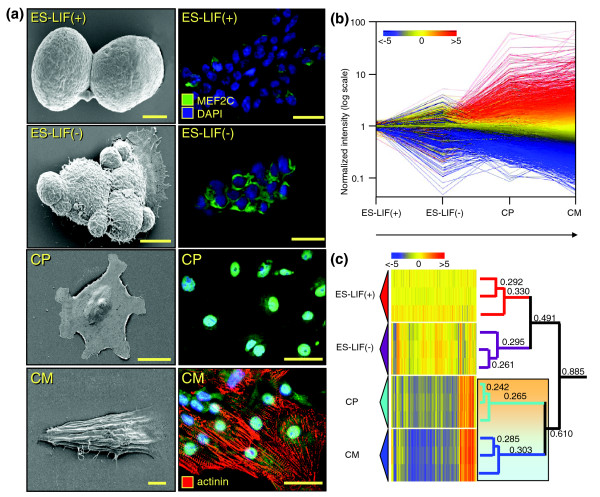
Phenotypic changes and transcriptome dynamism during cardiac stem cell differentiation. **(a) **Electron microscopy visualized morphological changes occurring during guided stem cell cardiogenesis (left column) with associated expression and distribution of the selected cardiac transcription factor MEF2C and the cardiac contractile protein α-actinin (right column). Cell stage is given in the top left corner of each panel with associated scale bars at the bottom right. First column: ES-LIF(+), 2.5 μm; ES-LIF(-), 5 μm; cardiopoietic cell (CP), 25 μm; cardiomyocyte (CM), 5 μm. All scale bars in the second column indicate 10 μm. Nuclei were counterstained with DAPI. **(b) **Transcriptional profiling of samples from each stage of stem cell-derived cardiomyocyte formation. Changes in gene expression were plotted on a semi-log scale graph using normalized intensity values as a function of the stage of differentiation. The color scale indicates increased expression (red), no change (yellow) and decreased expression (blue). Associated numbers indicate fold change, where red and blue indicate a respective minimum five-fold up- or downregulation in expression value. **(c) **Hierarchical clustering of changing genes during differentiation. The condition tree on right illustrates similarity of replicates within each stage. Numbers above branches are the calculated Euclidean distances between the two samples at the left termini. Smaller numbers indicate less dissimilarity between samples while higher numbers indicate an increase in dissimilarity. The shaded box identifies emergence of cardiac specficity (orange, CP) with transition to stem cell derived cardiomyocyte (cyan, CM). The color scale indicates relative changes in gene expression as described previously.

Pluripotency is a labile characteristic of embryonic stem cells amenable to specification by distinct inductive stimuli [9,22]. Here, to initiate cardiac commitment in undifferentiated stem cells, the recognized cardioinductive potential of the cytokine tumor necrosis factor (TNF)α-induced, endodermally derived paracrine factors was reduced to a collective cocktail, that is, bone morphogenetic protein (BMP), transforming growth factor (TGF)β, interleukin (IL)-13 (IL13), IL3, insulin-like growth factor (IGF1), vascular endothelial growth factor (VEGF), epidermal growth factor (EGF), fibroblast growth factor (FGF) and IL6 [18]. Cardiogenic cocktail-primed embryonic stem cells responded by structural metamorphosis and progressive up-regulation in canonical cardiac markers, with distinct phenotypes resolved by sequential field emission scanning electron microscopy (Figure 1a, left) and immunofluorescence (Figure 1a, right). Embryonic stem cells, initially maintained in the undifferentiated proliferative state in the presence of the mitogenic leukemia inhibitory factor [23], assumed a spheroid shape with high nuclear-to-cytoplasmic volumes, and lacked the cardiac sarcomeric protein α-actinin with marginally detectable cytosolic levels of the cardiac transcription factor myocyte enhancer factor 2C (MEF2C; Figure 1a). From this original state, mitogen removal initiated differentiation, characterized by a progressive decrease in the nuclear-to-cytoplasmic volume ratio and an increased expression of MEF2C accompanied by cytosolic-to-nuclear translocation (Figure 1a). Developmentally regulated nuclear import of cardiac transcription factors is indicative of definitive commitment to cardiac differentiation [19]. Accordingly, these intermediate cell types have been termed cardiopoietic stem cells [18]. Sustained nuclear import of MEF2C and formation of sarcomeres expressing cardiac α-actinin after 12 days identified mature, functional cardiomyocyte morphology. The degree of purity for derived progenitors and cardiomyocytes reached 85 ± 5% and 90 ± 5%, respectively (see Materials and methods). Interrogation of the developing transcriptome revealed 8,656 quality-filtered genes underlying guided cardiopoietic lineage specification, resolved into distinct groups of increasing, decreasing or unchanging profiles (Figure 1b). Concomitant with dynamic trends of lineage specification, each stage of cardiac differentiation demonstrated discrete molecular fingerprints revealed by unsupervised agglomerative clustering (Figure 1c). Gene sets were highly similar within, but significantly distinct between, stages of cardiac differentiation. Hierarchical categorization using Euclidean distance was used to measure differences between expression profiles to determine dissimilarity among replicates (Figure 1c). Unbiased confidence levels for these reproducible transcriptional profiles were assessed by bootstrapping, used to determine the accuracy of statistical estimates [24]. All distance measurements possessed a 100% confidence level and demonstrated increasing similarity towards the smaller, terminal branches of the condition tree. Small distances (≤0.33) reflected close association among replicate gene profiles, which were virtually inseparable at each stage of differentiation (Figure 1c). Larger Euclidean distances of 0.491 and 0.610 indicated greater dissimilarity between embryonic stem cells in the presence and absence of mitogen, as well as between cardiopoietic precursors and derived cardiomyocytes, allowing for separation of respective genomic fingerprints (Figure 1c). The largest measurement (0.885) reflected macroscopic differences between undifferentiated stem cells and lineage-specified populations (Figure 1c). Thus, discrete clustering of transcriptome dynamics during guided cardiogenesis genetically delimits precursor phenotype underlying cardiac confinement of stem cells.

### Tailored gene ontology directing cardiopoiesis

**Figure 2:**
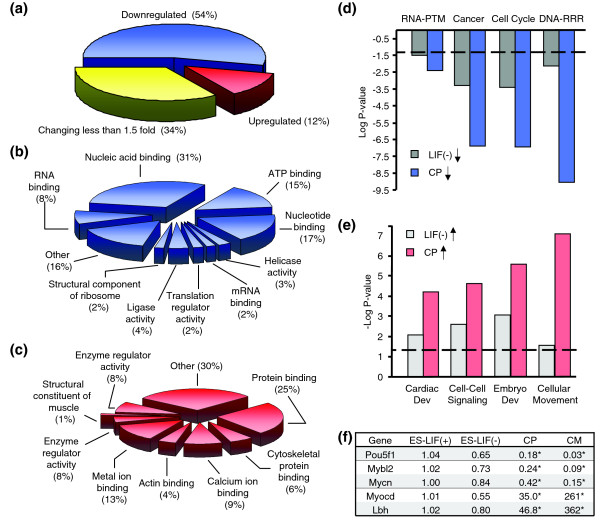
Enrichment analysis of functional groups within the stem cell-derived cardiopoietic transcriptome. **(a) **Approximately half of all expression profiles in cardiopoietic cells are downregulated while a third do not change more than 1.5-fold compared to unstimulated embryonic stem cells. Upregulated genes account for >10% of all genes. **(b, c) **Ontological analysis of downregulated and upregulated biological processes in cardiopoietic cells. **(d, e) **Identification of overrepresented canonical functions in cardiopoietic cells (CP) using Ingenuity Pathways Analysis (IPA) in downregulated and upregulated gene lists. Significance as determined by IPA was plotted as log *P *value for downregulated genes and -log *P *value for those upregulated to emphasize direction of change. The dashed line indicates the threshold where the *P *value = 0.05. Embryonic stem cells in the presence of mitogenic LIF were taken as baseline and significant functional enrichment in cardiopoietic cells are shown in comparison with stem cells cultured without LIF. **(f) **Gene validation using quantitative PCR. Candidate genes representing pluripotent (*Pou5f1*), oncogenic (*Mybl2*, *Mycn*) and cardiac (*Myocd*, *Lbh*) phenotypes were assayed by Taqman. Transcriptional profile changes were expressed as fold change relative to ES-LIF(+). CM, cardiomyocyte.

Restrictive quality filtering of the transcriptome to genes with dynamics exceeding a >1.5-fold change in cardiac precursors relative to undifferentiated embryonic stem cells yielded 1,069 (12%) and 4,632 (54%) genes up- and downregulated, respectively, with 2,955 (34%) transcripts changing by <1.5-fold (Figure 2a). Analyses of subthreshold genes below the 1.5-fold limit revealed no predominant functional overrepresentation within ontologically annotated families (data not shown). In contrast, genes identified as up- or downregulated beyond 1.5-fold unmasked overrepresented molecular functions in each gene set (Figure 2b,c). Genetic metabolism, identified by nucleotide binding, helicase and ligase activity, ribosomal structure, and translation regulator activity, was downregulated in cardiac precursors (Figure 2b). Alternative corroboration reported functional reductions in RNA post-translational modifications, oncogenic processes (for example, *Aurkb *and *Hmgb1*), cell cycling, and DNA replication, recombination and repair (Figure 2d). Decreased nucleotide metabolic machinery was paralleled by emergence of myogenic structural constituents, actin and calcium binding activities, and protein modification mechanisms regulating enzyme function (Figure 2c). Independent validation demonstrated that upregulated transcripts functionally overrepresented cardiovascular development, cell-to-cell signaling, embryonic development and cellular movement (Figure 2e). Collectively, this ontological switch indicates congruent genetic losses and gains that define a departure from oncogenicity associated with pluripotency towards acquisition of tissue-specificity and cardiopoietic elaboration. Gene chip and functional categorization analyses were verified by quantitative genetic amplification of markers for pluripotency (*Pou5f1*/*Oct4*), oncogenesis (*Mybl2*, *Mycn*) and cardiogenesis (*Myocd*, *Lbh*). *Pou5f1 *transcription, prototypical of pluripotent stem cells [25], was decreased as embryonic stem cells underwent differentiation (Figure 2f). Transcription of *Mybl2 *and *Mycn*, markers for neoplastic growth and tumor susceptibility [26,27], paralleled *Pou5f1 *expression and decreased as the cardiac program progressed (Figure 2f). In contrast, developmental expression of myocardial *Myocd *[28] and *Lbh *[29] genes increased during cardiac specification (Figure 2f). Thus, concomitant genetic streamlining with targeted induction of a focused transcriptome defines essential requirements for cardiopoietic lineage establishment.

### Cardiopoiesis-associated signaling cascades

**Figure 3:**
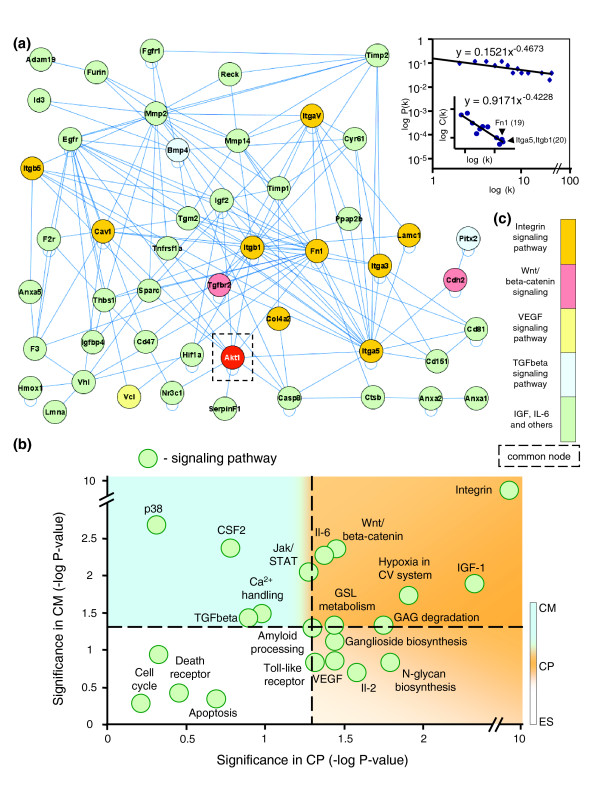
Cardiovascular development signaling network within cardiopoietic cells. **(a) **Genes identified in Table 1 integrate into a network suggesting non-stochastic tendencies with emergent scale-free properties (top right). Examples of hubs, with number of first neighbor connections in parentheses, are labeled on the clustering coefficient plot (top right, inset). **(b) **All upregulated genes in cardiopoietic cells analyzed for enriched functions were further mined to identify top supporting signaling cascades. Individual signaling pathways (green circles) were distributed according to significance during stem cell-derived cardiogenesis, indicating differences in pathway prioritization at discrete stages. The color scale at right indicates progression from embryonic stem cells (ES) through the cardiopoietic stage (CP) to stem cell-derived cardiomyocytes (CM), shown in counterclockwise fashion. CSF, colony stimulating factor; GAG, glycosaminoglycan; GSL, glycosphingolipid. **(c) **Cross-referencing the signaling cascades represented in (a) with all cardiopoietic pathways identified in (b) converge on integrin, WNT/β-catenin, VEGF, TGFβ and other (IGF, IL6) signaling cascades anchoring the procardiogenic network. A common node shared by these pathways, AKT, is outlined in (a).

Analysis of genes associated with the ontological 'Cardiac development' class in the specialized precursor transcriptome was composed of 65 upregulated genes (Table 1). Of these, 49 integrated into a cardiopoietic network (Figure 3a), while 16 did not possess curated interactions (Table 1). Inspection of network topology through degree and clustering coefficient distribution analysis suggested non-arbitrary architecture with hierarchical tendencies (Figure 3a). Bioinformatic investigation of underlying signaling pathways revealed individual overrepresented cascades, reported using cardiopoietic and cardiomyocyte significance estimates as respective co-ordinates in a Cartesian plot (Figure 3b). Cell cycle, death receptor and apoptosis cascades were examples of pathways with *P *values below significance threshold for both cardiopoietic cells and cardiomyocytes (Figure 3b, bottom left), in line with reported downregulation of genes required for cell proliferation and apoptotic processes in fully differentiated embryonic stem cell-derived cardiomyocytes [11]. In contrast, VEGF, IL2 and Toll-like receptor signaling were relevant at initiation of cardiac confinement, accompanied by amyloid processing, glycosphingolipid metabolism, glycosaminoglycan degradation, and N-glycan and ganglioside biosynthesis (Figure 3b, lower right). Integrin, WNT/β-catenin, IL6, IGF1 and cardiovascular hypoxia signaling pathways, initially prominent in cardiopoietic cells, maintained a significant presence in stem cell-derived cardiomyocytes (Figure 3b, top right), which began expressing genes involved in TGFβ, JAK/STAT, p38, granulocyte-macrophage colony stimulating factor/colony stimulating factor 2, and calcium signaling (Figure 3b, top left), in agreement with identified enrichment of p38 signaling and calcium handling [11]. A cross-section of signaling pathways with cardiac development revealed convergence of VEGF, integrin, WNT/β-catenin and TGFβ cascades, and connections involving IL6, IGF1 and JAK/STAT signaling (Figure 3c). Thus, discrete cascades anchor the molecular cardiopoietic network.

**Table 1:** Cardiopoietic cells demonstrate specific upregulation of genes involved in cardiovascular development

Gene name	GenBank ID	Fold change
*Actc	NM_009608	1.755
*Acvr1	NM_007394	2.531
*Adam19*	NM_009616	1.955
*Akt1*	M94335	2.518
*Amot	U80888	3.321
*Anxa1*	NM_010730	9.544
*Anxa2*	NM_007585	4.472
*Anxa5*	D63423	6.957
*Axl	AA500897	16.45
*Bmp4*	NM_007554	1.552
*Casp8*	BC006737	1.994
*Cav1*	AB029929	10.81
*Cd47*	NM_010581	2.265
*Cd81*	NM_133655	1.599
*Cd151*	U89772	1.597
*Cdh2*	BC022107	2.861
*Col4a2*	BC013560	16.98
*Ctsb*	M14222	4.591
*Cyr61*	NM_010516	6.394
*Ece1	AI551117	2.551
*Egfr*	AF277898	1.641
*F3*	BC024886	3.146
*F2r*	BQ173958	3.117
*Fgfr1*	M65053	1.807
*Fn1*	BC004724	2.536
*Furin*	NM_011046	1.858
*Has2	NM_008216	2.57
*Hif1a*	BB269715	2.586
*Hmox1*	NM_010442	1.728
*Id3*	NM_008321	1.75
*Igf2*	NM_010514	22.08
*Igfbp4*	NM_010517	10.46
*ltga3*	NM_013565	1.549
*ltga5*	BB493533	2.908
*Itgav*	AK003416	2.012
*Itgb1*	BM120341	2.642
*Itgb5*	NM_010580	2.542
*Lamc1*	BG066605	3.632
*Lmna*	NM_019390	4.11
*Ltbr	NM_010736	5.021
*Mixl1	AF154573	1.647
*Mmp2*	NM_008610	5.483
*Mmp14*	NM_008608	5.742
*Nf1	BB526552	1.542
*Nr3c1*	NM_008173	1.836
*Pitx2*	U80011	2.062
*Pou6f1	AK009674	1.647
*Ppap2b*	NM_080555	2.42
*Ppp3r1	NM_024459	1.704
*Reck*	NM_016678	2.305
*Sema3c	AK004119	3.763
*Serpinf1*	NM_011340	8.628
*Smo	AW55532	1.652
*Sparc*	NM_009242	5.953
*Sptbn1	BM213516	1.628
*Tgfbr2*	BG793483	13.3
*Tgm2*	BC016492	2.979
*Thbs1*	AI385532	12.43
*Timp1*	BC008107	4.251
*Timp2*	BF168458	40.6
*Tnfrsf12a	NM_013749	3.322
*Tnfrsf1a*	L26349	4.763
*Vcl*	NM_009502	3.886
*Vhl*	NM_009507	1.523
*Zfpm1*	AA014267	3.272

A total of 65 genes were upregulated with transition from a pluripotent embryonic stem cell into the cardiopoietic phenotype, 49 of which associated as an integrated network (Figure 3a). *Genes without curated interactions.

### Cardiopoietic network manipulation controls cardiogenic yield

**Figure 4:**
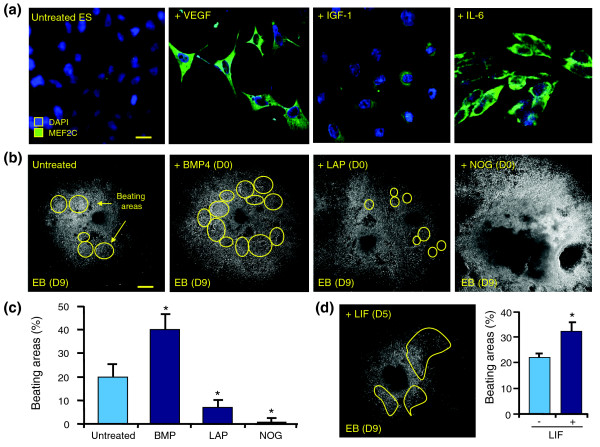
Biological validation of cardiogenic network. **(a) **LIF cultured stem cells were left untreated for 48 h after LIF withdrawal or were treated with VEGF, IGF1 or IL6. Changes in expression of the cardiac transcription factor MEF2C were revealed through immunocytochemistry. Nuclei were counterstained with DAPI. Scale bar: 10 μm. **(b) **Stem cell-derived embryoid bodies were observed for the formation of beating areas (yellow circles) at day 9 (D9) in untreated, BMP4, LAP and NOG supplemented conditions, with treatments beginning at day zero (D0). Scale bar: 1 μm. **(c) **Measurement of contractile area activity using Metamorph software. Data reported as mean number of beating areas ± SEM, *P < 0.05, *n *= 40-50 embryoid bodies. **(d) **Visualization of beating areas in embryoid bodies treated at day 5 (D5) with LIF, involved in JAK/STAT signaling (left). Evaluation of beating area as a percentage of total area occupied by embryoid body (right). Data reported as mean number of beating areas ± SEM, *P < 0.05, *n *= 40-50 embryoid bodies.

Consequences of targeting designated pro-cardiogenic components were investigated in isolated stem cells and differentiating embryoid bodies (Figure 4). While stimulating pathways absent from the identified cardiopoietic network had no effect on outcome (not illustrated), treatment of embryonic stem cells with VEGF, IGF1 and IL6, to prioritize charted signaling axes, increased expression of MEF2C (Figure 4a). Together with Nkx2-5 and GATA4 (data not shown), these pro-cardiac transcription factors were upregulated after growth factor supplementation, verifying association with cardiomyogenesis. To investigate effects of treatment at later developmental stages, stem cell-derived embryoid bodies were assessed for beating areas, which reflect emergence of electro-mechanical coupling (Figure 4b). BMP4, administered at day 9 of differentiation, increased the number of beating areas compared to untreated embryoid bodies (Figure 4b, left panels). Conversely, treatment with the TGFβ signaling cascade inhibitor latency-associated peptide (LAP) [30] significantly diminished the size and number of these areas at day 9, while alternative inhibition with the BMP4 antagonist NOG [31] abrogated the development of contractile foci (Figure 4b, right panels). On average, there was an approximately 20% increase in contractile regions within the embryoid body following BMP4 treatment, while addition of LAP decreased this number to <10% of the embryoid body. NOG treatment precluded contractile foci generation (Figure 4c). Investigation of the JAK/STAT pathway on cardiopoiesis was performed by adding leukemia inhibitory factor (LIF), which promoted beating area formation (Figure 4d). Thus, focused evaluation of individual network elements translated into changes in cardiogenic yield, validating the functional significance of the identified pro-cardiac scaffold.

### Cluster analysis reveals defined functional neighborhoods

**Figure 5:**
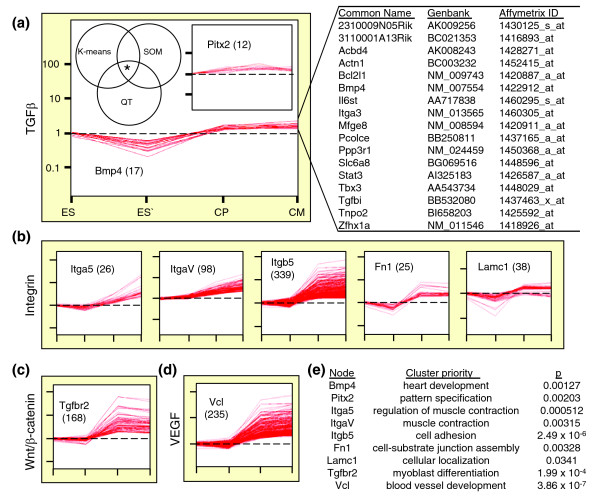
Nodal network anchors orchestrate coordinated recruitment of specialized ontological classes to secure a developmental theme. **(a) **Left: a five group K-means algorithm, 4 × 6 SOM, and QT filter were each used to independently dissect the transcriptome of embryonic stem cell (ES) derived cardiogenesis. Cardiopoietic (CP) network nodes previously identified were then used to guide cluster extraction. Bmp4 and Pitx2, members of the TGFβ pathway, are shown as examples. *Venn diagram of K-means, SOM and QT generated lists resolved clustered genes with correlated expression profiles (R = 0.95). For each set, gene (node) identity used to extract associated profiles is indicated, along with number of probes per cluster given in parentheses. Right: genes within the Bmp4 cluster. CM, cardiomyocyte. **(b) **Nodes belonging to the integrin cascade select discrete clusters with distinct trends. **(c, d) **Gene groups associated with Tgfbr2 and Vcl nodes that represent WNT/β-catenin and VEGF signaling, respectively. **(e) **Gene clusters organized functional neighborhoods with ontological priorities, with level of significance indicated on right.

Within the cardiopoietic network, integrin, Wnt/β-catenin, VEGF and TGFβ anchor cascades all contain specific genes used as foci for expression pattern segregation. Discrete correlated expression profiles within the transcriptome were refined by Venn diagram analysis to yield shared signature genes (Figure 5a and Additional data file 1). Bmp4 and Pitx2 are elements of the TGFβ cascade within the cardiopoietic network that coordinated organization of 17 and 12 gene profiles, respectively, into significantly correlated clusters (Figure 5a). Multiple genes that comprise integrin signaling within the network were queried separately and yielded unique gene lists with distinct trends (Figure 5b). Tgfbr2, a component of the Wnt pathway, distilled a core of 168 probesets (Figure 5c). Vcl integrates the VEGF cascade into the cardiopoietic network and here extracted 235 associated expression patterns (Figure 5d). Each cluster presented a significant ontological function upon analysis, with cardiac specification as the first, rank-ordered tissue-specific developmental process. Myoblast differentiation, regulation of muscle contraction, cellular localization/assembly, and vascular development were also prioritized within each cluster according to associated *P *values (Figure 5e). Therefore, specific functional properties were ascribed to each network node mapping respective contributions to the overall execution of cardiopoietic transformation of embryonic stem cells.

## Discussion

Embryonic stem cells are developmentally malleable [32], giving rise to highly specialized and discrete phenotypes crucial to the formative embryo. Specification through genomic tailoring of stem cell pluripotency involves parsing the massive transcriptional background and deploying necessary genetic instructions that drive commitment [33]. Since lineage segregation is governed by specific stimuli arising from a rich transcriptional landscape, mapping pathways directing distinct cellular fates is essential in identifying, engaging and driving selected developmental routes [34]. The paradigm of guided cardiogenesis used here provides a unique opportunity to dissect complex developmental processes underlying cardiopoiesis, essential for cardiomyocyte derivation from stem cells [18,35,36]. Using this paradigm in conjunction with *in silico *bioinformatics approaches, comparative genomic analyses uncovered a novel function-directed interactome connecting discrete genes that orchestrate cardiopoiesis. The identified multi-nodal transcriptome network establishes the cardiogenic gestalt, revealing targets for manipulation of cardiac fate that will expedite translational application [37-39].

Endogenous genetic flux through non-specific pluripotency primes stem cells for a variety of phenotypes at the cost of elevated genetic noise [40,41]. Successful navigation of this intricate genetic background is pivotal in developmental specification, requiring non-stochastic gene activation to support emerging phenotypes [42]. Systems biology approaches to analyze network randomness and propensity for hub formation [43] suggest a robust topology framing the transcriptome that underlies cardiopoiesis.

Specifically, the current work demonstrates formation of an integrated scaffold of genes activated during stem cell-derived cardiomyocyte procurement that sculpts a resilient cardiogenic transcriptome profile. The non-random presence and distribution of hubs, that is, nodes with high connectivity [44], indicates a switch where pluripotent stem cells are directed and constrained to a cardiac fate. While nonsignificantly changing genes represented heterogeneous ontological annotation distributions without any functional predispositions, inspection of downregulated transcripts demonstrated a controlled diminution of transcriptional chatter through reduction of genes associated with genetic metabolism. Furthermore, diminished DNA metabolism that accompanies differentiation of embryonic stem cells into cardiac precursors reflects the onset of specialization with loss of genomic variability. Indeed, timing changes and restriction of replication initiation has been reported for embryonic stem cell differentiation [45]. Transcriptional narrowing has been recently observed during stem cell differentiation for both nuclear transport machinery [19] and metabolic energetic remodeling [46]. Further streamlining, with specific upregulation of overrepresented pro-cardiogenic functions [35,47], ultimately secures execution of the cardiac program.

Gene or protein networks buttress pluripotency through integration of multiple pathways contributing to the final phenotype [48-51]. Here, distinct organization of signal pathways secured the cardiopoietic network. Integrins are cytoskeletal associated transmembrane glycoproteins that transduce extracellular signals and prominently anchor the transcriptome. Within cardiogenesis, the integrin cascade dictates formation of terminal myocardial structure [52]. WNT/β-catenin signaling transduces extracellular cues during development [53] and is the second significant cascade identified in the cardiopoietic network. Previous transcriptome analysis identified upregulation of negative regulators of WNT signaling [11]. Here, in guided cardiopoiesis, distinct effectors that feed into the WNT pathway were upregulated. Participation of this cascade in cardiogenesis is bimodal [54-56], and concomitant expression variations of inhibitors and potentiators may serve as a molecular rheostat indispensable for all types of lineage specification. In this capacity, the WNT family has been proposed to be transcriptional noise filters during differentiation [42]. The TGFβ cascade, connected to the cardiopoietic scaffold through BMP4, represents a source of potent pro-cardiac stimuli [23,57-59]. Transgenic models deficient in downstream signaling components of the TGFβ pathway, such as SMAD3 insufficient mice, exhibit cardiac developmental defects [60]. Furthermore, cell-tracing studies recently reported progenitors positive for the VEGF receptor FLK1 that gave rise to cardiovascular, endothelial and smooth muscle cell lineages [61,62], in line with the present identification of the VEGF signaling axis in the pro-cardiac transcriptome network. Cardiomyocyte development through IGF [55], and cardiac hypertrophy mediated by the IL6 signaling axis [63] are represented herein by single components belonging to their respective pathways. The inclusion of other cascades in the cardiopoietic network, also by single component representation, permits integration of lambent inputs from other pathways, lessening the rigidity of the transcriptome scaffold and allowing exogenous manipulability of the network without changing its fundamental architecture. Collectively, integration of discrete signaling pathways secures overall network functionality and, indeed, targeting nodes *in vitro *demonstrates discernible alterations in stem cell-derived cardiomyocyte yields.

Targeted node validation by independent treatment with different growth factors significantly increased the number of embryonic stem cells positive for cardiac transcription factors, indicating an engaged cardiogenic program. VEGF, upon binding to its cognate receptor on the surface of embryonic stem cells [64], is transduced through focal adhesion complexes containing vinculin [65], a significant node identified within the cardiopoietic network. This actin-binding, cytoskeletal protein is essential to cardiac development, as knockout models presented thin myocardial walls with compromised cardiac contractility along with diverse cardiac defects [66]. Similarly, interaction of IGF1 with the IGF1 receptor expressed on the plasma membrane of embryonic stem cells [67] increased the number of stem cells with an engaged cardiac program. Both AKT and IGF binding protein 4, elements of the IGF1 pathway essential to the cardiopoietic network, promote cell survival and proliferation, and affect organismal growth [55,68,69]. AKT is critical for directing hypertrophic myocardial responses to adaptive and maladaptive stimuli [70-73]. IL6 belongs to JAK/STAT/IL6 signal transducer (IL6ST)-dependent cytokines, and here increased cardiogenic engagement. This is supported by reports of modulated cardiogenesis in embryoid bodies through the JAK/STAT/IL6ST relay [74]. Conditional mutations of IL6ST, a component of the IL6 receptor complex, manifest cardiac defects, including ventricular thinning, right ventricular dilation, and significant size reductions in subpopulations of cardiomyocytes [63]. Furthermore, genetic ablation of IL6ST demonstrates a definitive role for the IL6 signaling axis in determination and maintenance of cardiac morphology [75]. Functionally, formation of contractile areas is a definitive endpoint indicating syncytial integration of developed cardiomyocytes. Treatment with BMP4, a cardiopoietic network ligand of the TGFβ cascade [76], distinctly increased beating areas, whereas antagonism using LAP or NOG precluded beating. Together, these observations reveal that the TGFβ signaling axis is embedded within the cardiopoietic network, supported by well characterized effects on cardiogenesis [23,77]. LIF treatment increased contractile foci, and exerts cardiogenic effects through the JAK/STAT/IL6ST signaling complex. Thus, the interactive transcriptome transduces pro-cardiac inputs, reflected through cardiogenic engagement and subsequent functional cardiomyocyte generation.

Network anchors within the emergent cardiovascular scaffold are part of extant transcriptome gene clusters that collectively foster distinct thematic climes [78]. As cellular identities manifest from embryonic stem cell origins, developmental programming is oriented through hubs that are part of an ontological collective that defines specific transcriptome neighborhoods and secures nascent phenotypes [79]. Furthermore, here collective ontological themes classifying hub-organized gene clusters are complementary and non-stochastic, demonstrated in this paradigm of cardiogenesis. In this way, the transcriptomic framework serves as a 'wireframe' that co-ordinates and unifies discrete developmental elements to ultimately realize full specification.

## Conclusion

Here, a manipulable, lineage-specifying genomic atlas was extracted from the pluripotent content of an embryonic source. Transcriptomic profile dissection of embryonic stem cells undergoing cardiopoietic transition isolated a dynamic intermolecular signaling scaffold unifying genetic crosstalk critical to cardiogenic yield. Functional interrogation of this focused network demonstrated treatment-dependent, bimodal responsiveness dictated by node and hub composition. A demonstrable, refined control of guided cardiogenesis by *in vitro *supplementation with exogenous growth factors efficiently accelerated the production of functional cardiomyocytes. In contrast, addition of network decelerants delayed cardiogenesis. Thus, access and identification of nodes within the cardiopoietic network is distinctly advantageous for procurement of an exogenous supply of cardiac cells. This circumvents limitations associated with a scarce endogenous pool, and expedites translation of *ex vivo *stem cell-derived cardiac-specified progeny as a regenerative therapeutic modality. Consolidation of node-organized functional transcript clusters secured developmental attunement through coordinated ontological neighborhoods that contained candidates promoting cardiac development. This paradigm of a defined gene network architecture, supportive of the cardiac progenitor phenotype, provides a diagnostic map to chart susceptible nodes that conversely may promote cardiomyocyte attrition with resultant cardiac dysfunctions. Critical rate-limiting hubs within such a framework can identify unexplored molecular etiologies that impact cardiac precursor lifespan or capacity for self-renewal, defining individual cardioprotective potential. Ultimately, this integrated approach maps a dynamic and interactive transcriptomic grid for definition, interrogation, and control of a discrete biological process.

## Materials and methods

### Stem cell culture and differentiation

Murine CGR8 embryonic stem (ES) cells were cultured without a feeder layer in 7.5% fetal bovine serum (FBS) in Glasgow's modified Eagle's medium (GMEM) as described [23]. Cells in the presence of LIF and after 48 h in a LIF-free environment were designated as ES-LIF(+) and ES-LIF(-), respectively. Subsequently, embryonic stem cells were placed in a cocktail containing 5 ng/ml BMP, 2.5 ng/ml TGFβ, 100 ng/ml IL-13, 100 ng/ml IL3, 50 ng/ml IGF1, 10 ng/ml VEGF, 2.5 ng/ml EGF, 10 ng/ml FGF and 100 ng/ml IL6. Cardiopoietic cells and cardiomyocytes derived from embryonic stem cells stimulated in this cocktail were maintained in culture using 3% FBS GMEM with 30 ng/ml of TNFα for 5 days and 20% FBS GMEM for 9 days, respectively [18]. Cells were subjected to confocal microscopy, assessing MEF2C, NKX2-5 and GATA4 nuclear translocation in cardiopoietic cells along with expression of α-actinin or myosin heavy chain in cardiomyocytes both prior to and after purification of derived cells using Percoll. The gradient was generated with dilution of a Percoll stock (Sigma-Aldrich, St. Louis, Missouri, USA) to densities of 1.09 and 1.07 g/ml, with 4 ml of the 1.07 density overlaying 3 ml of the 1.09 density. The interface of these two densities successfully yielded the cardiomyocyte population [19]. For cardiopoietic cells, the previous densities used for cardiomyocyte derivation were reduced by 0.02 g/ml [18]. Total RNA was harvested from ES-LIF(+), ES-LIF(-), cardiopoietic and cardiomyocyte samples for downstream microarray analysis.

### Scanning electron microscopy

Embryonic stem cells, cardiopoietic cells or derived cardiomyocytes were fixed with 1% glutaraldehyde and 4% formaldehyde in phosphate buffered saline (pH 7.2). Hypotonic sarcolemmal stripping using a 1% Triton X-100 solution exposed the nucleus prior to fixation [19]. Intact or stripped fixed cells were rinsed in phosphate buffered saline with 1% osmium, dehydrated with ethanol and dried in a critical point dryer. Samples were examined on a 4700 field-emission scanning microscope (Hitachi, Tokyo, Japan) after coating with platinum.

### Stem cell immunocytochemistry and embryoid body imaging

Embryonic stem cells, cardiopoietic cells and derived cardiomyocytes were fixed in 3% paraformaldehyde, permeabilized with 0.5% Triton X-100, blocked with 100% Superblock (Pierce, Rockford, Illinois, USA) and immunostained with primary antibodies specific for the cardiac transcription factor MEF2C (Cell Signaling Technology, Boston, Massachusetts, USA) and/or sarcomeric α-actinin (Sigma-Aldrich, St Louis, Missouri, USA), and corresponding ALEXA-labelled secondary antibodies (Molecular Probes, Carlsbad, California, USA) along with nuclear-staining 4'-6-diamidino-2-phenylindole (DAPI; Molecular Probes) [19]. Imaging was performed using a Zeiss laser scanning microscope 510 (Carl Zeiss, Jena, Germany) microscope. Additionally, after 48 h treatment of undifferentiated embryonic stem cells with 50 ng/ml IGF1, 10 ng/ml VEGF, or 100 ng/ml IL6 following LIF withdrawal, images were obtained and stored in TIF format with 10 distinct fields from at least 3 separate isolations for each experimental condition. Image evaluation of fluorescent intensity was performed using Metamorph (Sunnyvale, California, USA). Differentiated embryoid bodies, using the established hanging drop method [80], were treated at day 0 (D0) with 5 ng/ml BMP4, 25 ng/ml LAP, or 25 ng/ml NOG. Alternatively, 1,000 U/ml LIF was added at day 5 (D5) of differentiation. Prior to imaging at day 9 (D9), embryoid bodies were plated on gelatin-coated six well dishes with sequential timelapse images obtained at 5 Hz. Image sets were reconstituted in Metamorph to visualize beating areas, delineated for area measurement.

### Microarrays

To investigate transcriptome dynamics during guided cardiac differentiation of murine embryonic stem cells, total RNA was isolated at discrete timepoints using the Micro-to-Midi Total RNA Purification System (Invitrogen, Carlsbad, California, USA) as described [46]. Each condition was independently sampled three times for a total of twelve biological replicates. Double stranded complementary cDNA and labeled complementary cRNA were obtained from isolated total RNA, with the latter hybridized against the Mouse 430 2.0 GeneChip (Affymetrix, Santa Clara, California, USA). Arrays were scanned using an argon-ion laser, and data visualized using MAS 5.0 Affymetrix software to assess quality of hybridization. The dataset has been deposited at the Gene Expression Omnibus [81] as an update to series GSE6689.

### Expression analysis and gene/condition clustering

Gene expression data were analyzed using Genespring GX 7.3 (Agilent Technologies, Santa Clara, California, USA). All probesets were initially quality filtered for the pluripotent embryonic stem cell transcriptome (in the presence and absence of LIF) according to an established flag value, with values that are present (P), marginal (M) or absent (A) assigned to the marker [46]. To ensure that transcriptional changes were restricted to display gene profiles emerging during cardiac differentiation, data were further restricted to display genes demonstrating the present (P) and marginal (M) flag values in all three replicates for the cardiopoietic stage, and the present (P) flag value in all stem cell-derived cardiomyocyte samples. Next, samples were filtered according to background noise levels to remove genes expressing signals below threshold. The final gene list was delimited according to statistically relevant changes using one-way ANOVA, *P *< 0.05 with the Benjamini and Hochberg false discovery rate as multiple testing correction. Hierarchical dendrograms were used to establish the molecular fingerprints for each stage, and were generated using the Pearson coefficient statistic (*r*) applied to determine correlation between gene pairs in each condition as follows:

(1) $$r=\frac{\sum_{i=1}^{n}(A_{i}-\bar{A})(B_{i}-\bar{B})}{\sqrt{\left(\sum_{i=1}^{n}{\left(A_{i}-\bar{A}\right)}^{2}\right)\left(\sum_{i=1}^{n}{\left(B_{i}-\bar{B}\right)}^{2}\right)}}$$

Above, (*A*) and (*B*) are respective sample means for genes *A*_*i *_and *B*_*i *_for sample (*i*) out of the total number of samples (*n*), with standard deviation terms for *A*_*i *_and *B*_*i *_used as denominator. Condition clustering was performed to determine sample similarity using Euclidean distance as a measure of sample 'nearness'. The formula for calculating distance (*D*):

(2) $$D=\sqrt{\frac{1}{n}\sum_{i=1}^{n}{\left(A_{i}-B_{i}\right)}^{2}}$$

The square of the difference in expression levels between gene A (*A*_*i*_) and gene B (*B*_*i*_) in sample (*i*) are divided by the total number of samples (*n*), of which the square root is taken to obtain distance (*D*). The clustering derived from distance calculation was further validated by bootstrapping, a conventional statistical resampling technique [24].

### Taqman assays

RNA (1 μg) was reverse transcribed into cDNA using a high capacity cDNA archive kit (Applied Biosystems, Foster City, California, USA) and assayed using Taqman gene expression assays for *Pou5f1/Oct4 *(Mm00658129_gH), *Mybl2 *(Mm00485340_m1), *Mycn *(Mm00476449_m1), *Myocd *(Mm00455051_m1) and *Lbh *(Mm00522505_m1), prototypical markers of pluripotency, oncogenesis and cardiogenesis. Samples were loaded onto an optical 96-well plate for polymerase chain reactions performed using an ABI 7900HT Fast Real Time System with cycling parameters set for a 15 s, 95°C duplex denaturing step followed by primer annealing/extending for 1 minute at 60°C per cycle for 40 cycles. Relative fold change was determined using the 2^-ΔΔC^_T _method [82] with pluripotent embryonic stem cells as baseline, normalized to *Gapdh *expression.

### Enrichment analysis of functional categories

To examine overrepresented functions within the final up- and downregulated filtered gene lists, Expression Analysis Systematic Explorer (EASE version 2.0) [83] was used. Gene lists were submitted as text files using GenBank accession identifiers and ontology annotations for 'Molecular function' were analyzed by linking, through EASE, to the online Database for Annotation, Visualization, and Integrated Discovery [84]. For 'Molecular function', the population total (8,821) is the group of annotations available for the Mouse 430 2.0 GeneChip. Population hits are defined as the genes for each 'Molecular function' sub-classification that are identifiable. List totals indicate annotations (out of the population total) that are available from user-submitted lists for 'Molecular function', and list hits identify annotations belonging to specific groups within 'Molecular function' within the user-submitted list. Each category under 'Molecular function' had specifically associated genes, and in some instances, genes were assigned to more than one functional category. Significance was determined by Fisher's exact test and Bonferroni correction for multiple category comparisons (*P *< 0.05) and top functions were reported as a percentage of list totals, with remaining functions classified as 'other' for both up- and downregulated gene lists.

### Network analysis

Using an established network analysis program, Ingenuity Pathways Analysis [85], molecular interactions were examined in the cardiopoietic stage. One way ANOVA-delimited gene lists used in enrichment analysis were studied using the Ingenuity Pathways Knowledge base to identify, using a right-tailed Fisher's exact test, overrepresented canonical functions and signaling pathways at different timepoints during cardiogenic stem cell differentiation. The Institute for Systems Biology Cytoscape 2.2 software [86] was applied to provide data regarding network topology in addition to visualizing relationships. Gene interactions identified by Ingenuity were deconstructed according to pairwise interactions, and reformatted for use in Cytoscape 2.2. Basic network analyses, including degree distribution and clustering coefficient distribution determination, were performed, providing statistical measures of cardiopoietic network architecture.

### Cluster analysis

Quality filtered genes were recursively and separately analyzed by K-means, self-organizing map (SOM) and quality threshold (QT) clustering. Group size for K-means was set to a maximum of 5 clusters, while a 4 × 6 array was specified for SOM. For QT analysis, Pearson correlation was set at 0.95. Each of the three analyses produced distinct transcript aggregates, and cross-reference by Venn diagram highlighted genes consistently segregating with selected network nodes. Discrete expression profile groups were bioinformatically mined to uncover organized functional neighborhoods delimited by cluster oriented developmental themes. Hypergeometric *P *values for ontological assignations were calculated as shown:

(3) $$p=\frac{1}{\left(\begin{array}{c} u\\ m \end{array}\right)}\sum_{i=k}\left(\begin{array}{c} m\\ i \end{array}\right)\left(\frac{u-m}{n-1}\right)$$

The summation notation above yields the probability (p) of overlap that corresponds to (*k*) or more genes that exist between gene lists (*m*) and (*n*) when randomly sampled from a universe containg (*u*) genes.

## Abbreviations

BMP, bone morphogenic protein; DAPI, 4'-6-diamidino-2-phenylindole; EASE, Expression Analysis Systematic Explorer; EGF, epidermal growth factor; ES, embryonic stem; FBS, fetal bovine serum; FGF, fibroblast growth factor; GMEM, Glasgow's modified Eagle's medium; IGF1, insulin-like growth factor; IL, interleukin; IL6ST, IL-6 signal transducer; LAP, latency associated peptide; LIF, leukemia inhibitory factor; MEF2C, myocyte enhancer factor 2C; QT, quality threshold; SOM, self organizing map; TGF, transforming growth factor; TNF, tumor necrosis factor; VEGF, vascular endothelial growth factor.

## Authors' contributions

RSF, AB, CPT and AT contributed to the design of the study. RSF performed bioinformatics involved in this study. AB carried out cell culture and immunocytochemistry. CPT did electron microscopy. RSF, AB, and CPT analyzed the data. RSF, AB, CPT and AT prepared the manuscript. All authors have read and approved the final version of this manuscript.

## Additional data files

The following additional data are available with the online version of this paper. Additional data file 1 is an Excel spreadsheet listing gene identities within node organized clusters.

## Supplementary Material

Additional data file 1 Cluster analysis yielded genes associated with network node anchors that possessed correlated and discrete expression profiles, with a few anchors sharing a single group. Furthermore, each transcript cluster fostered a prevalent ontological specification, detailed in Figure 5. Click here for file
